# Human Milk Casein and Whey Protein and Infant Body Composition over the First 12 Months of Lactation

**DOI:** 10.3390/nu10091332

**Published:** 2018-09-19

**Authors:** Zoya Gridneva, Wan J. Tie, Alethea Rea, Ching Tat Lai, Leigh C. Ward, Kevin Murray, Peter E. Hartmann, Donna T. Geddes

**Affiliations:** 1 School of Molecular Sciences, M310, The University of Western Australia, Crawley, Perth, WA 6009, Australia; ash.tie@uwa.edu.au (W.J.T.); ching-tat.lai@uwa.edu.au (C.T.L.); peter.hartmann@uwa.edu.au (P.E.H.); donna.geddes@uwa.edu.au (D.T.G.); 2 Centre for Applied Statistics, The University of Western Australia, Crawley, Perth, WA 6009, Australia; alethea.rea@uwa.edu.au; 3 School of Chemistry and Molecular Biosciences, The University of Queensland, St. Lucia, Brisbane, QLD 4072, Australia; l.ward@uq.edu.au; 4 School of Population and Global Health, The University of Western Australia, Crawley, Perth, WA 6009, Australia; kevin.murray@uwa.edu.au

**Keywords:** casein, whey, protein, breastfeeding, infant, body composition, bioelectrical impedance spectroscopy, ultrasound skinfolds, human milk, calculated daily intakes, lactation

## Abstract

Human milk (HM) influences infant feeding patterns and body composition (BC). This small proof-of concept longitudinal study investigated relationships between infant/maternal BC and HM casein, whey and total protein during the first 12 months of lactation. BC of breastfeeding dyads (*n* = 20) was measured at 2 (*n* = 15), 5 (*n* = 20), 9 (*n* = 19), and/or 12 (*n* = 18) months postpartum with ultrasound skinfolds (infants) and bioimpedance spectroscopy (infants/mothers). Proteins concentrations and 24-h milk intake were measured and calculated daily intakes (CDI) determined. Higher maternal weight, body mass index, fat-free mass, fat-free mass index, and fat mass index were associated with higher concentration of whey protein (*p* ≤ 0.034, *n* = 20). There were no associations between infant BC and concentrations of all proteins, and CDI of whey and total protein. Higher CDI of casein were associated with lower infant fat-free mass (*p* = 0.003, *n* = 18) and higher fat mass (*p* < 0.001), fat mass index (*p* = 0.001, *n* = 18), and % fat mass (*p* < 0.001, *n* = 18) measured with ultrasound skinfolds. These results show a differential effect of HM casein on development of infant BC during the first year of life, suggesting that there is a potential to improve outcome for the infant through interventions, such as continuation of breastfeeding during the first 12 months of life and beyond, which may facilitate favourable developmental programming that could reduce risk of non-communicable diseases later in life.

## 1. Introduction

An epidemic of childhood obesity and the associated non-communicable diseases (NCD), such as diabetes and cardiovascular disease are of increasing international concern [1]. Increasingly, data suggests the lifelong risk of NCD could be modified through early programming effects on obesity and adiposity [2,3]. It is evident that breastfed infants are at 15–20% reduced risk of obesity and obesity related disease later in life [4,5]. Whilst the protective mechanisms of breastfeeding are not fully understood [6], the development of body composition (BC) in early life [7], composition of human milk (HM) [8,9,10], and infant breastfeeding patterns and behavior [11,12,13] are all known to play an important role in the programming of these health outcomes.

An increased risk of obesity has been associated with both rapid weight gain [14] and the elevated protein content in infant formulas [9,15]. Compared with 9 g/L in term HM (6–12 g/L, week 10/12) [16], protein content ranged from 12 to 19 g/L in infant formulas and from 16 to 27 g/L in follow-up formulas [15]. Thus, limiting protein intake from infant foods could be an effective strategy in reduction of childhood obesity, leading to the development of lower protein formulas to mimic growth rates of HM fed infants [15]. HM is recognized as the best form of nutrition for optimal growth and development of the human infant and as such is species specific in composition. The stark compositional differences between HM and formula have been implicated in the differences in weight and BC between breastfed and formula-fed infants and decreased risk of later obesity for breastfed infants [8,9].

The protein content of HM, which is low yet highly bioavailable, appears to play a key role in infant growth [9] and might provide a rationale for the reduced risk of being in rapid-growth trajectory [17] and for the lower fat-free mass (FFM) and higher fat mass (FM) and percentage FM (%FM) of breastfed infants compared with formula-fed [18] during early infancy. In term infants, the protein intakes from HM (three months, boys: 6.4 ± 1.2 g/day; girls: 5.8 ± 1.0 g/day) were found to be almost half that of formula (boys: 11.4 ± 2.0 g/day; girls: 10.5 ± 1.8 g/day), and at three and six months differences in protein intake and weight gain were associated with disproportionate gain in FM and FFM, resulting in %FM difference between the groups [8]. These variations suggest a self-regulatory mechanism of milk intake (MI), which may be in part driven by HM components [10] that are associated with feeding frequency (FFQ) [19].

Despite MI and protein intakes being associated with breastfed infants’ weight and FFM gain [8] very few comprehensive longitudinal HM protein intake studies are available through the exclusive breastfeeding and weaning periods. Also, there has been no investigation of the effect of either concentrations or daily intakes of specific HM fractions—such as casein and whey—on infant BC, yet these are highly variable between breastfeeding dyads. Daily intakes of HM components are the true reflection on what infant receives, unlike the concentrations, which can be misleading, especially if the milk intake is inadequate or if mixed feeding is present. 

The protective role of HM and breastfeeding may be attributable to the effect of specific protein fractions on development of infant BC. Thus, the aim of this longitudinal study was to investigate relationships of concentrations and daily intakes of HM casein, whey and total protein with anthropometrics and BC of healthy term breastfed infants and their mothers during first 12 months postpartum. Further exploration of relationships of infant 24-h MI and FFQ with HM proteins was carried out.

## 2. Materials and Methods

### 2.1. Study Participants

Breastfed infants (*n* = 20; 10 males, 10 females) of English-speaking, predominantly Caucasian, mothers of higher social-economic status from a developed country were recruited from the community, primarily from the West Australian branch of the Australian Breastfeeding Association. Inclusion criteria were: healthy singletons, gestational age ≥37 weeks, exclusively breastfed [20] at 2 and 5 months, and maternal intention to breastfeed until 12 months. Exclusion criteria were: infant factors that could potentially influence growth and development of BC, maternal smoking, and low milk supply. All mothers provided written informed consent to participate in the study, which was approved by The University of Western Australia Human Research Ethics Committee (RA/1/4253, RA/4/1/2639) and registered with the Australian New Zealand Clinical Trials Registry (ACTRN12616000368437).

### 2.2. Study Session

Measurements were made when the infants were 2 and/or 5, 9, and 12 months of age. Participants visited our laboratory at King Edward Memorial Hospital for Women (Subiaco, Perth, WA, Australia) for up to four monitored breastfeeding sessions between March 2013 and September 2015. At each study session, the infant was weighed pre-feed, and then the mother breastfed her infant. Infant bioelectrical impedance spectroscopy (BIS) measurements were made pre-feed, unless impractical, then they were taken post-feed [21]. Ultrasound skinfold and anthropometric measurements were made post-feed. Clothing was removed for the measurements except for a dry diaper and a sleeveless shirt.

Maternal weight, height, and BIS measurements were recorded. Small (1−2 mL) pre- and post-feed milk samples were collected into 5 mL polypropylene vials (Disposable Products, Adelaide, SA, Australia) from the breast/s that infant was fed from and samples were frozen at −20 °C for biochemical analysis. Current feeding frequency (FFQ) of the infants was self-reported by mothers.

### 2.3. Anthropometric Measurements

Infants weight was determined before breastfeeding using Medela Electronic Baby Weigh Scales (±2.0 g; Medela Inc., McHenry, IL, USA). Infant crown-heel length was measured once to the nearest 0.1 cm using non-stretch tape and a headpiece and a footpiece, both applied perpendicularly to the hard surface. Infant head circumference was measured with a non-stretch tape to the nearest 0.1 cm.

Maternal weight was measured using Seca electronic scales (±0.1 kg; Seca, Chino, CA, USA). Height was self-reported by participants or measured against a calibrated marked wall (accuracy ±0.1 cm). 

Infant and maternal body mass index (BMI) were calculated as kg/m^2^.

### 2.4. Body Composition with Bioelectrical Impedance Spectroscopy

The methods for measuring maternal and infant BC with BIS using Impedimed SFB7 bioelectrical impedance analyser (ImpediMed, Brisbane, QLD, Australia) as well as age-specific equations used for calculations of infant BC parameters during this study have been published previously [22]. The within participant coefficient of variation (CV) for maternal %FM was 0.21% [23]. Within participant CV for infant resistance measurements at 50 kHz (R_50_) was 1.5% [21].

### 2.5. Ultrasound Skinfold Measurements

The method for measuring infant skinfolds using the Aplio XG ultrasound machine (Toshiba, Tokyo, Japan) with a 14-8 MHz transducer (PLT-1204BX) and sterile water-based ultrasonic gel (Parker Laboratories Inc., Fairfield, NJ, USA) as well as the equations for calculations of infant BC parameters during this study have been published previously [22,24]. Briefly, single ultrasound scans of four anatomical sites (biceps, subscapular, suprailiac and triceps) were performed on the left side of the body with minimal compression. At all tome points, infant BC was calculated with both, 2-skinfolds (US 2SF: triceps, subscapular) and 4-skinfolds (US 4SF: biceps, subscapular, suprailiac and triceps).

### 2.6. Body Composition Indices

The indices of height-normalized BC were calculated for mothers and infants: FM index (FMI) was calculated as FM/length^2^, and FFM index (FFMI) was calculated as FFM/length^2^; both expressed as kg/m^2^ [25].

### 2.7. 24-h Milk Intake and Feeding Frequency

Infant 24-h MI was measured by mothers using the 24-h milk production (MP) protocol, weighing infants at home with the Medela Electronic Baby Weigh Scales pre- and post each breastfeed during a 24-h period plus one breastfeeding, and recording amounts of HM (g) consumed by the infant (including expressed HM if any) [26]. 24-h MI was determined as previously described with potential underestimation of 3–10% [26] and FFQ (meals per 24-h) was recorded [27]. 24-h MI was measured at three time points: between 2 and 5 (4.0 ± 1.3) months, when MI is shown to be stable [27], and within 2 weeks of 9 (9.4 ± 0.3) and 12 (12.2 ± 0.4) months. Given that measuring 24-h MI is not always practical, particularly at the later stages of lactation, mothers were also asked to estimate how frequently the infant fed, and self-reported (SR) the typical time between the meals (e.g., each 2 h) during the week prior to the study session as a proxy measure of FFQ.

### 2.8. Calculated Daily Intakes of Human Milk Proteins

24-h MI values from the 24-h MP, and casein, whey and total protein concentrations (pooled pre-/post-feed) from samples taken at the study sessions were used for determination of calculated daily intakes (CDI). These CDI were considered representative of a typical daily intake due to the absence of significant short-term (weekly) [28] and circadian [29,30] variations in HM protein concentrations during the established lactation. 

### 2.9. Sample Preparation

Prior to further analysis, HM samples were thawed for 2 h at room temperature, mixed on Intelli-Mixer RM-2M (ELMI, Riga, Latvia) at 50 revolutions per min in “UU” mode for 15 s, then, after gentle inversion (three times), aliquoted into 1.5 mL tubes (Sarstedt, Numbrecht, Germany). Pre- and post-feed samples were pooled for measuring casein and whey and total protein. Milk samples were defatted by centrifugation at room temperature in a Beckman Microfuge 11 (Aberdon Enterprise Inc., Elk Grove Village, IL, USA) at 10,000× *g* for 10 min and removing the fat layer by clipping it off together with the top of the tube [31]. Skim HM was used for measuring protein concentrations. Standard assays were adapted for and carried out using a JANUS workstation (PerkinElmer, Inc., Waltham, MA, USA) and measured on EnSpire (PerkinElmer, Inc., Waltham, MA, USA).

### 2.10. Human Milk Fractions

Casein and whey proteins were separated by the method described by Kunz and Lonnerdal [32] and Khan et al. [30]. Protein concentrations (total protein, casein, and whey proteins) were measured using the Bradford Protein Assay adapted from Mitoulas et al. [29]. Recovery of protein was 100.6 ± 5.2% (*n* = 5) with a detection limit of 0.031 g/L and an inter-assay CV of 7.8% (*n* = 18). Casein-whey ratio was calculated as follows:

*Casein* − *whey ratio* = casein concentration/whey protein concentration. (1)

### 2.11. Statistical Analyses

Data for this analysis came from the longitudinal study, the details of which have been described previously [22]. Descriptive statistics are reported as mean ± standard deviation (SD) (range); model parameters as estimate ± SE (standard error).

During this longitudinal study infants were measured at four time points (2 and/or 5, 9 and 12 months). An approximate sample size was calculated using the ‘F tests–Linear multiple regression: Fixed model: *R*^2^ increase’ option in G*Power [33] as if this was a cross-sectional study with equal numbers at each time. Allowing four predictors (three for age comparisons), α = 0.05 and 14 participants (56 sample points = 14 participants × 4 time points) would give the study power of 0.80 to detect an effect size of 0.15. This approach was selected, as there is no closed form expression suitable for the calculation of sample sizes for this research design [34], with the consideration that longitudinal study design is more powerful. Recruitment of participants at the 5-months point was introduced, as many mothers would not commit to a study that required breastfeeding to 12 months, when approached at 2 months. As a result, required number of participants was increased to 20 in order to maintain predicted power; this also addressed issues relating to missed visits. Missing data was dealt with using available case analysis.

The analyses for systematic differences in concentrations and CDI of adipokines at different months after birth used linear mixed model with age as effect factor and participant as a random factor. Differences between each month were analysed using general linear hypothesis tests (Tukey’s all pair comparisons).

Relationships between: (a) maternal BC and protein concentrations/CDI, and (b) protein concentrations/CDI and infant BC, (c) protein concentrations and breastfeeding parameters (24-h MI/FFQ), and (d) FFQ and CDI of proteins were analysed using linear mixed effects models. Each protein concentration/CDI or infant BC measure/index was considered separately as the response variable, and each model contained fixed effects of infant age (months), a predictor (maternal BC measure/index, protein concentration/CDI and breastfeeding parameters (24-h MI/FFQ)) and an interaction between infant age and predictor, as well as a random intercept per participant. If the interaction was not significant results were reported for the same model fitted without the interaction to assist in understanding the nature of the relationship between the predictor and outcome.

To investigate if there were differences by sex, relationships between infant characteristics and protein concentrations were also analysed using linear mixed effects models accounting for age and sex and an interaction between infant age and predictor, as well as a random intercept per participant.

Relationships between CDI of proteins measured between 2 and 5, and at 9 and 12 months after birth and changes (Δ) in infant BC and anthropometric parameters between the time points were analysed using linear regression models.

Owing to the large number of comparisons, a false discovery rate adjustment [35] was performed on associated subgroupings of results to the interaction *p*-value if it was less than 0.05 or to the main effect *p*-value. The adjusted significance levels are reported in Results and Tables and set at the 5% level otherwise. Statistical analysis was performed in R 3.1.2 [36]. Additional packages were used for linear mixed effects models (nlme, lme4, and car) [37,38,39], intra-class correlations (irr) [40], Tukey’s all pair comparisons (multcomp) [41] and graphics (ggplot2) [42].

## 3. Results

### 3.1. Subjects

Twenty-two infants were recruited; two infants (one male, one female) were excluded from the study after the 2-months visit (commenced weaning; personal circumstances). One female infant weaned after the 5-months visit and was not followed up further; 19 remaining infants were breastfed at 9 months; 17 infants continued to breastfeed at 12 months. Out of 18 infants measured at 12 months, 16 infants (89%) still continued to breastfeed; one male infant ceased breastfeeding 2 weeks before the 12-month appointment and one female infant stopped at 10 months after birth. 

Therefore, overall six infants missed one study session and one infant missed two study sessions. Five of these infants were not recruited until 5 months, one infant did not attend the study session at 9 months and two did not attend the study session at 12 months. 

Overall, 80 measures were expected however some were missing, specifically: infant weight (*n* = 9); infant BC parameters measured with US 2SF, and maternal age, weight, height, BMI and BC parameters measured with BIS (*n* = 10); infant head circumference (*n* = 11); infant length, BMI and BC parameters measured with US 4SF, concentrations of casein, whey and total protein (*n* = 12); infant BC parameters measured with BIS (*n* = 13); self-reported FFQ (*n* = 20). Missing data also occurred due to difficulties with conducting 24-h MI measurements at later stages of lactation. The following measurements from the 60 expected were missing: FFQ from 24-h MP (*n* = 26), 24-h MI and CDI of casein, whey and total protein (*n* = 27). Missing data were spread across the time points (Table 1).

Participant demographic characteristics collected at the start of the study are presented in Table 2, anthropometric and breastfeeding characteristics measured at the four study sessions are presented in Table 1. The more detailed determinants of maternal and infant BC as well as description of longitudinal changes in infant and maternal BC and breastfeeding parameters, and the associations between them have been reported previously [22].

### 3.2. Breastfeeding Parameters and Human Milk Proteins

HM protein concentrations and CDI at 4 time points are detailed in Table 3. CDI of casein, whey and total protein, 24-h MI, and both SR and MP FFQ decreased across the lactation (see Table 4 for estimates and significances). 

### 3.3. Maternal Body Composition and Human Milk Proteins

**Figure 1:**
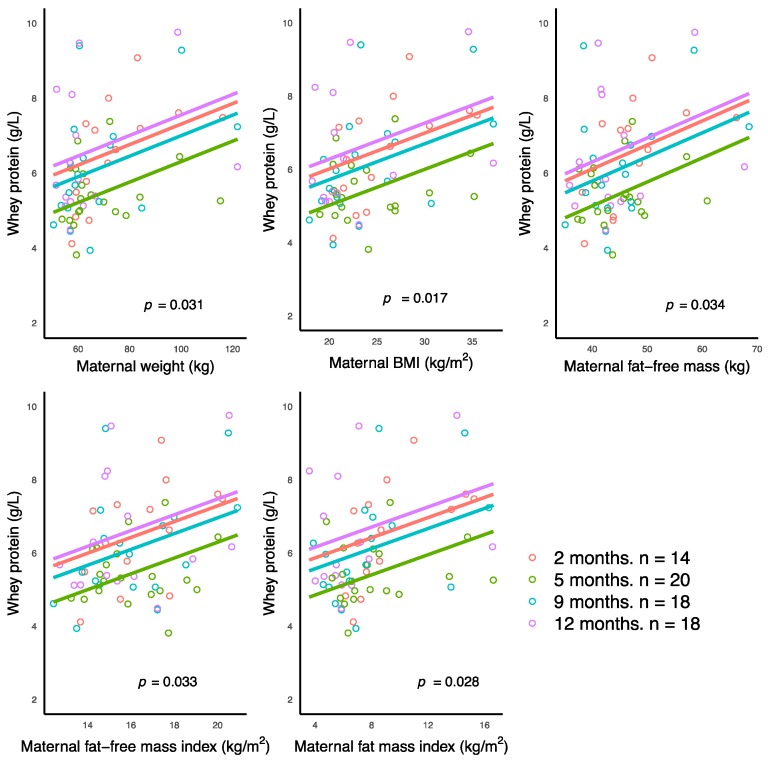
Significant positive associations between concentrations of human milk whey protein and maternal anthropometrics and body composition parameters measured with bioelectrical impedance spectroscopy. Lines represent linear regression and grouped by the month of lactation.

Higher maternal characteristics and BC measures (weight, BMI, FFM, FFMI, and FMI) were associated with higher whey protein concentrations (Figure 1).

No associations were seen between maternal characteristics and casein–whey ratio, total protein and casein concentrations, and CDI of all proteins after adjusting for the false discovery rate (see Table A1 for estimates and significances). 

### 3.4. Infant Body Composition and Concentrations of Proteins

No significant associations between concentrations of proteins and infant characteristics were seen after adjusting for the false discovery rate (see Table A2 for estimates and significances).

When infant sex was included in the models, significant differences were found for infant head circumference, weight, FFM (US 2SF, 4SF, and BIS) and FFMI (US 4SF and BIS), but none of the models reported an effect of either total protein, whey protein and casein concentrations or whey-to-casein ratio (details are not reported).

### 3.5. Infant Body Composition and Calculated Daily Intakes of Proteins

**Figure 2:**
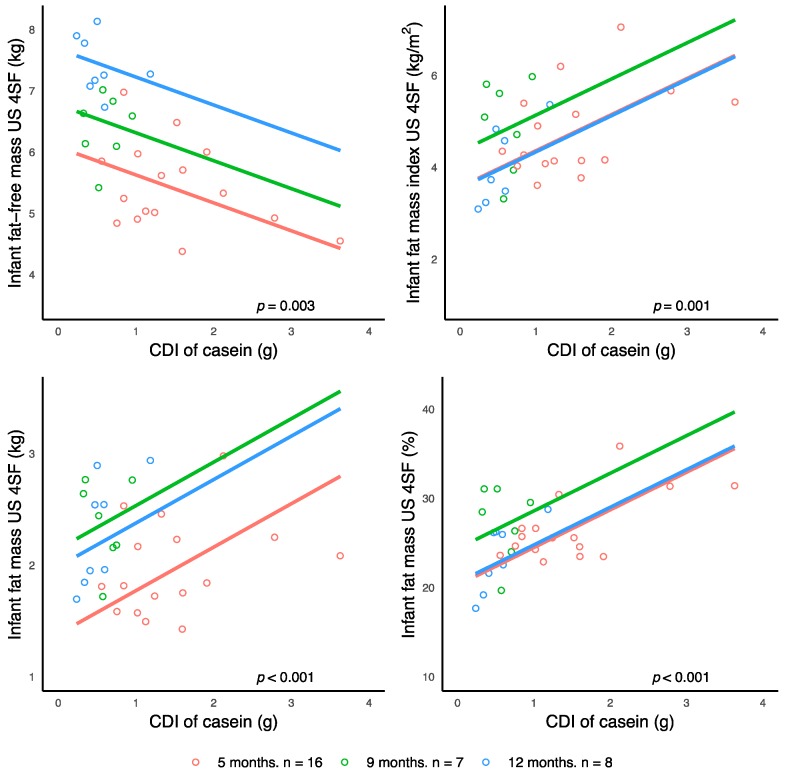
Significant associations between calculated daily intakes (CDI) of human milk casein and infant body composition parameters measured with ultrasound four-skinfolds. Lines represent linear regression and grouped by the month of lactation.

Higher CDI of casein were associated with lower infant FFM (US 4SF) and increased FM (US 4SF), %FM (US 4SF), and FMI (US 4SF) after adjusting for the false discovery rate (Figure 2).

No associations were seen between infant characteristics and both CDI of total protein and whey protein (see Table A3 for estimates and significances).

### 3.6. Breastfeeding Parameters and Human Milk Proteins

No significant associations were seen between concentrations of proteins and 24-h MI, 24-h MP FFQ, and SR FFQ after adjusting for the false discovery rate (see Table A4 for estimates and significances).

**Figure 3:**
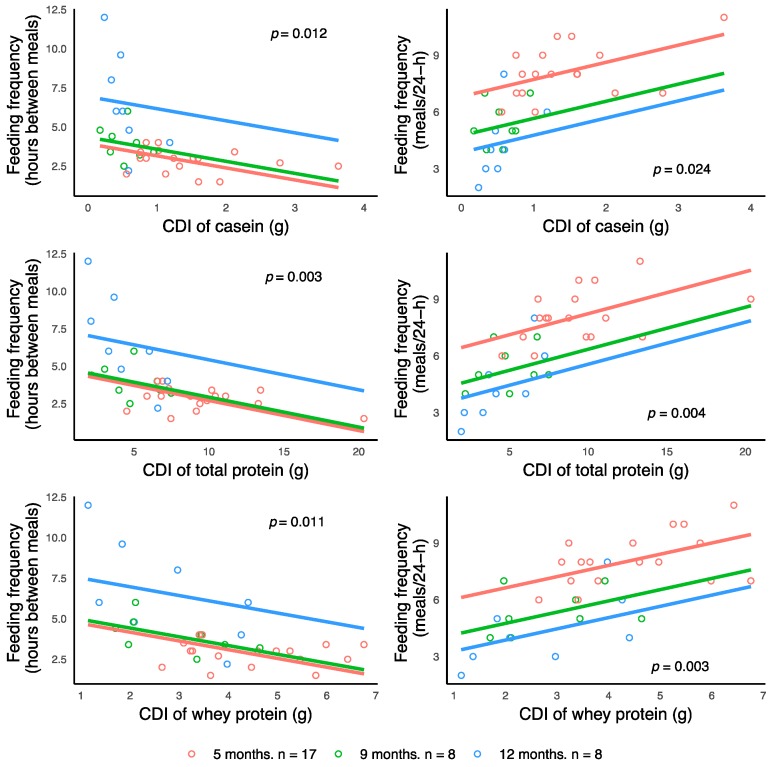
Significant associations between infant feeding frequency (self-reported feeding frequency (hours between meals) or 24-h milk production feeding frequency (meals per 24-h)) and calculated daily intakes (CDI) of casein and total and whey protein. Lines represent linear regression and grouped by the month of lactation.

Higher FFQ (both, SR and 24-h MP) were associated with higher CDI of all, casein, whey and total protein (Figure 3) after adjusting for the false discovery rate (see Table A4 for estimates and significances).

### 3.7. Changes in Infant Characteristics and Calculated Daily Intakes of Human Milk Proteins

After adjusting for the false discovery rate, no significant associations were seen at any time points between changes in infant BC (Δ) between the time points and CDI of either total protein, casein or whey protein (see Table A5, Table A6 and Table A7 for estimates and significances).

## 4. Discussion

**Figure 4:**
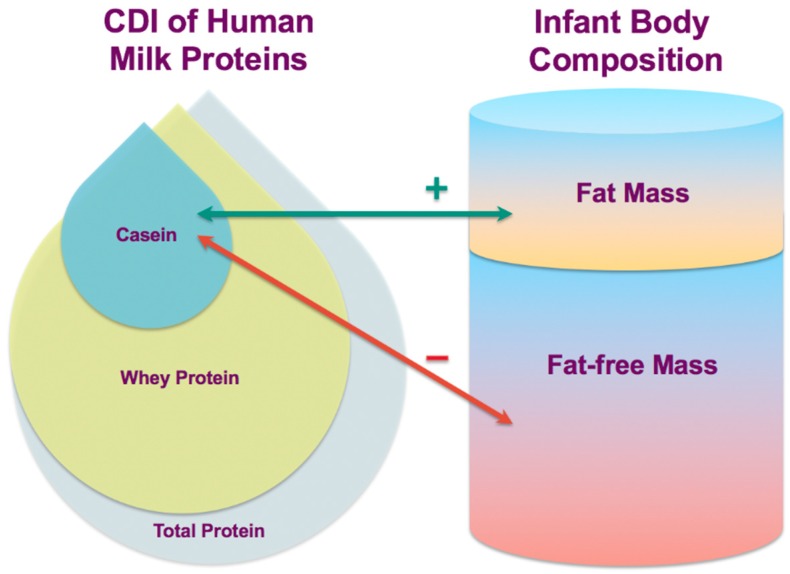
Possible lactocrine programming of the infant body composition during the first year of life as researched. Green arrow indicates positive associations of calculated daily intakes (CDI) of casein with measured body composition parameters and red arrow—negative associations.

A dose–response relationship between breastfeeding duration and childhood obesity has been previously confirmed [43] and this longitudinal study provides more knowledge on the complex mechanisms by which breastfeeding and HM may influence infant BC and confer some degree of protection from obesity. In this small proof-of-concept study, calculated daily intakes of HM casein have been associated with the development of infant BC and are differentially related to infant FM and FFM (Figure 4). Furthermore, infant FFQ was associated with higher CDI of casein, whey and total protein, emphasizing the critical role of HM and breastfeeding in programming of infant appetite control and growth in the first year of life.

Within the normal developmental context of breastfeeding we found that concentrations of total protein (approximately 11 g/L) were not associated with infant anthropometrics or BC during first 12 months of lactation (Section 3.4; Table A2). However, in formula-fed infants high protein formulas (15–19 g/L) are related to accelerated growth trajectory with greater weight gain [15], while breastfed infants display reduced lean body mass and increased adiposity during the early infancy compared to formula-fed [18]. Also in contrast to our results, higher HM protein concentrations at four to eight weeks postpartum were related to higher infant BMI at 12 months but not weight gain or BC [44]. These contrasting results are likely due to the differences in protein composition of formulas and shorter breastfeeding durations. Our study focused on women who breastfed for 12 months and therefore is more reflective of normative development of BC of the breastfed infant.

To account for variation in infant MI we calculated the CDI of total protein and found no association with infant anthropometrics or BC during first 12 months of lactation (Section 3.5; Table A3). Whilst volume of HM is known to be related to infant growth rate [29,45,46], it has been shown that infants consuming higher daily intakes of protein at various times during first 12 months have higher weight and FFM [8,47,48]. These studies however included formula-fed infants and concentrated on differences between breastfeeding and formula feeding by calculating combined protein intakes from HM/formula and solids. Our study showed multiple positive associations between CDI of total protein between two and five months and changes in infant BC, but statistical significance did not remain after correction for multiple comparisons (Table A5). One recent study measured protein intakes from HM during the first three months in two groups of breastfed infants, with higher and lower weight gain [10] and found protein intakes were not different between the groups. It therefore remains to be elucidated if total protein intake in breastfed infants does impact BC development and larger longitudinal trials are required to do this.

Notwithstanding the absence of infant dietary data, we found that higher CDI of HM casein are associated with both lower infant lean body mass and higher adiposity (Section 3.5; Table A3), with associations strengthening at the later months of lactation. Casein is present in HM at a very low concentrations, compared with other species [49] and is not only a source of amino acids and trace elements (calcium and phosphorus) for the infant [50] but also breaks down to the bioactive peptides that have an array of functions including antimicrobial, gastrointestinal, immuno-modulating and opioid effects [51]. Indeed, increased CDI of casein was also related to increased FFQ. Previously we have shown that higher FFQ was related to lower FFM and higher 24-h MI and adiposity [22]. Furthermore, we have also found higher casein-whey ratios of HM are associated with shorter gastric emptying time in a cross-sectional cohort of exclusively breastfed infants [52]. Casein-whey ratios also interacted with feed volumes, with higher casein-whey ratios associating with faster gastric emptying of smaller feed volumes and slower gastric emptying of larger feed volumes [52]. Thus, HM casein may be modulating the development of infant BC via gastric emptying and breastfeeding frequency that in turn influences infant MI.

CDI of HM whey protein produced similar associations with infant BC to CDI of casein; negative with lean mass and positive with adiposity (Table A3) however, correction for multiple comparisons eliminated significance. We have found previously that higher HM whey protein concentrations are associated with larger post-feed stomach volumes in exclusively breastfed infants [52], and may therefore have an effect on infant BC, in combination with casein, via modulation of breastfeeding patterns. Studies in animal models indicate that whey from bovine milk may promote the growth of the soft (and adipose) tissue [53]. Furthermore, infants fed whey-dominant formula had higher weight-for-age and BMI-for-age z-scores at four months compared with breastfed infants despite the protein content being reduced to match HM [54], although it should be noted that these differences could be explained by the lower feed volumes in the breastfed group. In contrast infants fed a whey- and casein-dominant formula displayed no differences in BC or anthropometrics at four months of age [55], compared to breastfed infants. Another study has reported greater fat deposition in males and greater gains in lean mass in females fed whey-dominant formula during first three months compared to breastfed infants [56]. In this study, we did not see any effect of infant sex on relationships of either casein or whey protein concentrations with infant anthropometrics or BC. Larger longitudinal studies that focus on the array of HM whey proteins and sex-specific analysis may resolve these conflicting results.

We have examined the casein and total whey proteins of HM however, the HM whey fraction contains proteins that remain soluble in the liquid portion after precipitation of caseins—such as α-lactalbumin, lactoferrin, lysozyme, and secretory IgA [57]—as well as various hormones, enzymes, and binding proteins [49]. Many of these proteins have not been studied in relation to infant growth and BC and may either act synergistically or in an antagonistic manner. Administered bovine and HM lysozyme [58,59] have been linked to increased weight gain in preterm infants. Furthermore, high levels of lactoferrin in bovine formula was associated with lower weight gain rate in healthy term female infants during first two months after birth compared with control [60]. Interestingly, α-lactalbumin-enriched formula did not result in any differences in weight gain, weight-for-age and weight-for-length z-scores compared with breastfed infants or infants fed standard formula at four months of age [61]. This suggests that α-lactalbumin, a rich source of essential amino acids, may play optimal nutritive but not necessarily programming role in infant growth. It is yet to be determined whether the proportions of the most abundant whey proteins impact growth and BC development, in particular lysozyme, which may act via promotion of the colonization of infant gut by the infant-type human-residential bifidobacteria [62] and via change in gut microbiome composition through both, detrimental microbe reduction and beneficial microbe enrichment as shown in porcine model [63]. 

The number of studies showing associations between maternal characteristics and HM components is increasing. We found that higher whey protein concentrations are related to higher maternal weight, BMI, and both fat and lean body mass throughout the first 12 months of lactation (Section 3.3, Table A1). This represents a potential pathway by which the protein composition of HM may be altered and potentially improved via optimal maternal BC which may impact infant development. The absence of an association with HM casein concentration suggests it is not modifiable, and reflects the intrinsic synthesis of casein in the breast [64] as opposed to movement of whey components from the maternal circulation into the milk [65]. Whilst we did not find any associations between maternal characteristics and total protein concentrations, total protein has previously been positively related to maternal %FM [23] and BMI [66,67,68,69]. Other studies have shown no associations [70] or a negative association [71]. Larger longitudinal studies would help to clarify these associations.

Breastfeeding frequency is likely a reflection of appetite regulation of the infant [72] and is highly variable between breastfeeding dyads [27]. Recently we have shown increased FFQ was associated with increased 24-h MI, and both of these breastfeeding parameters were related to higher infant adiposity [22]. Both methods that we used to measure infant FFQ have some limitations, especially self-reported FFQ which has been shown to be biased towards reporting higher numbers of feeds in infants that feed more frequently in comparison with 24-h MP FFQ [22], which is again limited to one measure at the time point of data collection. Nevertheless, positive relationships between FFQ (which were also associated with higher FM accretion) and CDI of HM proteins casein in particular (Section 3.6; Table A4) help us to assemble the possible pathways of the mechanisms of infant BC regulation. These results emphasize the importance of including these measures in order to elucidate the mechanisms by which HM components affect infant growth and BC and the degree by which the mother influences HM composition and volumes.

Daily intakes may be a more relevant factor than concentrations of HM components for examining the nutritional physiology of the breastfed infant. In this study, daily intakes were calculated and are considered representative of a typical daily intake due to the absence of significant within-feed [23], short-term (weekly) [28], and circadian [29] variations in HM protein concentrations, including casein and whey [30], during the established lactation. Furthermore, measurement of 24-h milk intake by test-weighing is an accurate [73] and reproducible procedure from within two to four days to within one to two weeks between measurements [45,74,75,76]. Although no dietary data was collected in this study, the evidence is emerging that protein from dairy/milk sources may have different and/or more potent effects on infant growth and adiposity than the vegetable and meat proteins probably due to the bioactive factors present in milks [77,78]. The major source of milk protein in this study was HM, as no formula was used by mothers. Furthermore, differences in protein intake between breastfed and formula-fed infants during first 12 months are mainly attributed to a lower intake of protein from HM, not to a difference in protein intake from solid foods [48].

The strength of this proof-of-concept study is that infants were breastfed on demand up to 12 months and the broad range of adiposity levels among the mothers. The limitations are the small number of participants, the small number of 24-h MP at 9 and 12 months and the absence of infant dietary data between 5 and 12 months of age. Our population was term healthy fully-breastfed singletons from predominantly Caucasian mothers of higher social-economic status therefore, our results may not be applicable to dyads from other backgrounds.

## 5. Conclusions

The results of this small proof-of-concept study provide more evidence that HM impacts infant developmental programming and show differential associations between HM casein intakes and development of infant fat and fat-free mass during the first 12 months of life. Thus, the continuation of breastfeeding past the exclusive breastfeeding period may facilitate developmental programming that could potentially reduce risk of obesity and NCD later in life.

## Figures and Tables

**Table 1:** Participant anthropometric and breastfeeding characteristics throughout 12 months of lactation.

	2 Months	5 Months	9 Months	12 Months
Characteristics	Mean ± SD	Mean ± SD	Mean ± SD	Mean ± SD
	(Range)	(Range)	(Range)	(Range)
**Mothers**				
	***n* = 14**	***n* = 20**	***n* = 18**	***n* = 18**
Weight (kg)	78.8 ± 19.3 ^a^	70.1 ± 17.8	63.0 ± 10.0	64.2 ± 17.3
	(57.5–116.2)	(53.7–115.3)	(50.4–121.9)	(51.4–121.9)
BMI (kg/m^2^)	27.2 ± 5.5	24.8 ± 5.0	22.7 ± 3.9	23.9 ± 5.9
	(20.4–35.5)	(19.0–35.2)	(17.9–37.2)	(18.2–37.2)
**Infants**				
	***n* = 14**	***n* = 20**	***n* = 19**	***n* = 18**
Sex (M/F)	9 M/6 F	10 M/10 F	10 M/9 F	9 M/9 F
Age (months)	2.04 ± 0.14	5.16 ± 0.22	9.22 ± 0.27	12.26 ± 0.28
	(1.87–2.33)	(4.77–5.47)	(8.83–9.77)	(11.63–12.67)
Length (cm)	58.1 ± 1.9	64.8 ± 2.3	71.7 ± 1.9	73.6 ± 3.2
	(54.2–60.0)	(60.5–69.5)	(66.0–74.0)	(69.0–78.5)
Weight (kg)	5.630 ± 0.660	7.431 ± 1.134	8.836 ± 0.975	9.650 ± 0.618
	(4.420–7.400)	(5.808–9.510)	(6.675–10.095)	(7.165–11.085)
BMI (kg/m^2^)	16.6 ± 1.2	17.6 ± 1.9	17.7 ± 1.7	17.8 ± 0.9
	(14.5–18.1)	(14.9–20.4)	(14.2–20.2)	(13.7–19.2)
Head circumference (cm)	39.7 ± 1.6	42.1 ± 1.5	45.6 ± 1.7	46.6 ± 1.7
	(37.0–42.0)	(40.0–45.9)	(43.0–48.5)	(44.2–49.5)
**Breastfeeding Characteristics**				
		***n* = 17**	***n* = 8**	***n* = 8**
24-h milk intake (g)	n/a ^b^	818.8 ± 204.9	478.3 ± 154.0	451.1 ± 215.7
		(498–1185)	(300–775)	(255–795)
		***n =* 17**	***n =* 8**	***n =* 9**
24-h feeding frequency (MP)	n/a ^b^	8.1 ± 1.4	5.4 ± 1.3	4.4 ± 2.1
		(6–11)	(4–7)	(2–8)
	***n =* 11**	***n =* 19**	***n =* 17**	***n =* 13**
Feeding frequency (SR)	2.3 ± 0.4 ^c^	2.8 ± 0.8	3.7 ± 1.2	5.4 ± 2.9
	(1.5–3.0)	(1.5–4.0)	(2.0–6.0)	(2.2–12.0)

^a^ Data are mean ± SD and ranges. ^b^ Milk intake and feeding frequency as meals per 24-h was determined from 24-h milk production (MP) measured between 2 and 5 months (presented at 5 months here) and within 2 weeks of 9 and 12 months; n/a, not applicable. ^c^ Maternal self-report (SR) of feeding frequency at the time of the visit as a typical time between meals (e.g., each 2 h). BMI, body mass index. M/F, male/female.

**Table 2:** Participant demographic characteristics at the start of the study.

Characteristics *n* = 20	Mean ± SD (Range)
Maternal age (years)	33.3 ± 4.7 (24–44) ^a^
Maternal height (cm)	167.4 ± 7.4 (150–181)
Parity	2.3 ± 0.9 (1–4)
Infant sex (Male/Female)	10/10
Infant birth weight (kg)	3.486 ± 0.498 (2.660–4.455)
Infant gestational age (weeks)	39.4 ± 1.3 (37.6–43.0)

^a^ Data are mean ± standard deviation (SD) and ranges.

**Table 3:** Human milk proteins presented as concentrations and 24-h intakes at months after birth ^a^.

	2 Months	5 Months	9 Months	12 Months
Components	Mean ± SD	Mean ± SD	Mean ± SD	Mean ± SD
	(Range)	(Range)	(Range)	(Range)
**Concentrations ^b^**				
	***n =* 15**	***n =* 20**	***n =* 19**	***n =* 15**
Total protein (g/L)	11.03 ± 1.40 ^c^	11.90 ± 4.31	9.69 ± 1.12	10.72 ± 2.84
	(7.60–12.32)	(7.93–24.16)	(7.25–14.96)	(5.89–16.80)
Casein (g/L)	1.24 ± 0.24	1.51 ± 0.44	1.11 ± 0.38	1.07 ± 0.35
	(0.69–1.57)	(0.78–3.45)	(0.49–2.00)	(0.65–1.87)
Whey protein (g/L)	6.44 ± 1.62	5.43 ± 0.90	5.43 ± 0.93	7.61 ± 1.85
	(4.12–9.08)	(3.82–7.38)	(3.94–9.40)	(4.49–9.76)
Casein-whey ratio	0.21 ± 0.07	0.28 ± 0.05	0.21 ± 0.08	0.16 ± 0.09
	(0.10–0.31)	(0.13–0.73)	(0.07–0.38)	(0.09–0.36)
**CDI ^d^**				
		***n =* 17**	***n =* 8**	***n =* 8**
Total protein (g)	n/a ^e^	9.19 ± 3.82 ^d^ (4.51–20.34)	5.24 ± 1.84	4.18 ± 2.11
			(2.18–7.48)	(1.93–7.23)
Casein (g)	n/a	1.45 ± 0.82 (0.56–3.63)	0.60 ± 0.23	0.54 ± 0.34
			(0.17–0.95)	(0.24–1.19)
Whey protein (g)	n/a	4.23 ± 1.14 (2.65–6.76)	3.02 ± 1.11	2.78 ± 1.34
			(1.70–4.64)	(1.15–4.40)

^a^ Milk components’ concentrations and 24-h components’ intakes are presented grouped by the month after birth. ^b^ Concentrations as measured at 2, 5, 9, and 12 months. ^c^ Data are mean ± SD and ranges. ^d^ Calculated daily intakes (CDI) of proteins were calculated between 2 and 5 months (presented at 5 months here) and within 2 weeks of 9 and 12 months. ^e^ n/a, not applicable.

**Table 4:** Differences by infant age/lactation duration (between time points) within measured human milk proteins and breastfeeding parameters ^a^.

Changes in Characteristics between Time Points	Months after Birth						
	5 and 2 Months	9 and 2 Months	12 and 2 Months	9 and 5 Months	12 and 5 Months	12 and 9 Months	*p* Overall
**Milk Components**							
	***n =* 15**	***n =* 13**	***n =* 10**	***n =* 18**	***n =* 14**	***n =* 15**	***n =* 20**
ΔTotal protein (g/L)	0.57 (0.80) ^b^	−0.52 (0.81)	0.08 (0.86)	−1.08 (0.75)	−0.49(0.80)	0.60 (0.81)	0.59 ^c^
	0.89	0.92	1.00	0.46	0.93	0.88	
ΔCasein (g/L)	**0.38 (0.15) ^d^**	0.87	1.00	**0.002**	**0.043**	0.89	**0.010**
	**0.047**	−0.11 (0.15)	−0.01 (0.16)	**−0.50 (0.14)**	**−0.39(0.15)**	0.11 (0.15)	
ΔWhey protein (g/L)	**−1.06 (0.36)**	−0.36 (0.36)	0.13 (0.39)	0.69 (0.33)	**1.19(0.36)**	0.49 (0.36)	**0.006**
	**0.016**	0.75	0.099	0.15	**0.005**	0.52	
ΔCasein-whey ratio	**0.11 (0.03)**	−0.01 (0.03)	0.01 (0.03)	**−0.11 (0.03)**	**−0.10 (0.03)**	0.01 (0.03)	**0.002**
	**0.004**	1.00	1.00	**<0.001**	**0.006**	0.98	
**Breastfeeding Characteristics**							
	***n =* 11**	***n =* 11**	***n =* 7**	***n =* 17**	***n =* 13**	***n =* 13**	***n =* 19**
ΔFeeding frequency (SR) ^e^	0.46 (0.53)	**1.40 (0.54)**	**3.14 (0.58)**	0.94 (0.46)	**2.69 (0.50)**	**1.75 (0.51)**	**<0.001**
	0.82	**0.045**	**<0.001**	0.17	**<0.001**	**0.003**	
				***n =* 7**	***n =* 7**	***n =* 7**	***n =* 10**
ΔFeeding frequency (MP) ^f^	n/a ^g^	n/a ^g^	n/a ^g^	**−2.81 (0.49)**	**−3.71 (0.46)**	−0.90 (0.52)	**<0.001**
				**<0.001**	**<0.001**	0.19	
				***n =* 7**	***n =* 7**	***n =* 7**	***n =* 9**
Δ24-h milk intake (g) ^f^	n/a	n/a	n/a	**−325 (64)**	**−376 (64)**	−52 (69)	**<0.001**
				**<0.001**	**<0.001**	0.73	
**CDI of Milk Components**							
				***n =* 7**	***n =* 7**	***n =* 7**	***n =* 9**
ΔTotal protein (g) ^f^	n/a	n/a	n/a	**−3.81 (0.83)**	**−4.09 (0.83)**	−0.28 (0.86)	**<0.001**
				**<0.001**	**<0.001**	0.94	
ΔCasein (g) ^f^	n/a	n/a	n/a	**−0.91 (0.26)**	**−0.91 (0.26)**	−0.002 (0.31)	**<0.001**
				**0.005**	**0.005**	1.00	
ΔWhey protein (g) ^f^	n/a	n/a	n/a	**−1.52 (0.37)**	**−1.49 (0.37)**	0.03 (0.39)	**0.002**
				**<0.001**	**<0.001**	1.00	

^a^ Systematic differences in the measured variables between different months after birth were calculated using general linear hypothesis test (Tukey’s all pair comparisons). ^b^ Data are parameter estimate and standard error of estimate and *p*-value. ^c^ Overall *p*-value is associated with age as reported in linear mixed model. ^d^ Bold text indicates significant difference (*p* < 0.05) between two time points or overall. ^e^ Feeding frequency was self-reported (SR) by mothers at the time of the visit as an average time between meals (e.g., each 2 h). ^f^ 24-h milk intake and feeding frequency as meals per 24-h was measured at 24-h milk production (MP) and daily intakes (CDI) calculated between 2 and 5 months (presented at 5 months here) and within 2 weeks of 9 and 12 months. ^g^ Results are not presented for impractical combinations; n/a, not applicable.

## Appendix A

**Table A1:** Associations between maternal characteristics and human milk proteins.

	2 Months		5 Months		9 Months		12 Months		*p*-Value		
Maternal Predictor	Intercept (SE)	Slope (SE)	Intercept (SE)	Slope (SE)	Intercept (SE)	Slope (SE)	Intercept (SE)	Slope (SE)	Predictor	Infant Age (Months)	Interaction
	***n =* 14**		***n =* 20**		***n =* 18**		***n =* 15**		***n =* 20**		
**Concentration of Whey Protein (g/L)**											
Weight (kg)	4.58 (0.96) ^a^	0.027 (0.013)	3.58 (0.91)	0.027 (0.013)	4.27 (0.90)	0.027 (0.013)	4.82 (0.89)	0.027 (0.013)	**0.031 ^b^**	0.003	0.26 ^c^
BMI ^d^ (kg/m^2^)	4.05 (1.09)	0.098 (0.041)	3.06 (1.04)	0.098 (0.041)	3.76 (1.03)	0.098 (0.041)	4.32 (1.01)	0.098 (0.041)	**0.017**	0.002	0.25
FFM ^d^ (kg)	3.55 (1.44)	0.064 (0.030)	2.56 (1.39)	0.064 (0.030)	3.23 (1.39)	0.064 (0.030)	3.74 (1.40)	0.064 (0.030)	**0.034**	0.004	0.39
FFMI ^d^ (kg)	2.97 (1.71)	0.216 (0.102)	1.98 (1.66)	0.216 (0.102)	2.64 (1.66)	0.216 (0.102)	3.16 (1.66)	0.216 (0.102)	**0.033**	0.004	0.43
FM ^d^ (kg/m^2^)	5.53 (0.60)	0.040 (0.020)	4.51 (0.55)	0.040 (0.020)	5.21 (0.54)	0.040 (0.020)	5.79 (0.53)	0.040 (0.020)	0.048	0.002	0.24
FMI ^d^ (kg/m^2^)	5.32 (0.64)	0.138 (0.063)	4.31 (0.59)	0.138 (0.063)	5.02 (0.58)	0.138 (0.063)	5.61 (0.56)	0.138 (0.063)	**0.028**	0.002	0.25
	**n/a ^e^**		***n* = 17**		***n =* 7**		***n =* 8**		***n =* 18**		
**CDI of Total Protein (g)**											
FM (%)	n/a ^e^	n/a ^e^	12.40 (4.28)	−0.090 (0.128)	5.30 (4.54)	0.019 (0.145)	−3.27 (4.47)	0.325 (0.153)	0.59	<0.001	0.046
**CDI of Whey Protein (g)**											
FFM (kg)	n/a	n/a	5.45 (2.24)	−0.002 (0.050)	−0.90 (2.54)	0.091 (0.057)	9.37 (4.76)	−0.155 (0.152)	0.82	<0.001	0.030

^a^ Parameter estimate ± SE; effects of predictors taken from linear mixed effects models that accounted for month after birth and an interaction between month after birth and predictor with a random effect per participant; if the interaction is not significant parameter estimates are taken from a model with no interaction. ^b,c^ Results are presented only for interactions or predictors with raw *p*-values < 0.05; after the false discovery rate adjustment, the interaction/predictor *p*-values were considered to be significant at <0.048 for concentration of whey protein (indicated by the bold text), at <0.05 for concentrations of total protein and casein, casein-whey ratio and CDI of casein (none are significant, data not shown), at <0.046 for CDI of total protein and at <0.030 for CDI of whey protein (none are significant). ^d^ BMI, body mass index; FFM, fat-free mass; FFMI, fat-free mass index; FM, fat mass; FMI, fat mass index. ^e^ CDI were measured between 2 and 5 months (presented at 5 months here) and within 2 weeks of 9 and 12 months; n/a, not applicable.

**Table A2:** Associations between concentrations of human milk proteins and infant characteristics.

	2 Months		5 Months		9 Months		12 Months		*p*-Value		
Predictor (Concentration, g/L)	Intercept (SE)	Slope (SE)	Intercept (SE)	Slope (SE)	Intercept (SE)	Slope (SE)	Intercept (SE)	Slope (SE)	Predictor	Infant Age (Months)	Interaction
	***n =* 13**		***n =* 20**		***n =* 18**		***n =* 13**		***n =* 20**		
**Infant Fat-Free Mass Index with Bioelectrical Impedance Spectroscopy (kg/m^2^)**											
Total protein	4.67 (0.29) ^a^	−0.034 (0.022)	5.66 (0.30)	−0.034 (0.022)	6.82 (0.28)	−0.034 (0.022)	7.48 (0.28)	−0.034 (0.022)	0.032 ^b^	0.070	0.55 ^c^
	***n =* 13**		***n =* 19**		***n =* 18**		***n =* 15**		***n =* 20**		
**Infant Fat Mass with Ultrasound 4 Skinfolds (%)**											
Casein-whey ratio	23.70 (1.36)	9.09 (4.62)	23.40 (1.70)	9.09 (4.62)	24.00 (1.28)	9.09 (4.62)	21.90 (1.33)	9.09 (4.62)	0.046	0.21	0.23

^a^ Parameter estimate ± SE; effects of predictors taken from linear mixed effects models that accounted for month after birth and an interaction between month after birth and predictor with a random effect per participant; if the interaction is not significant parameter estimates are taken from a model with no interaction. ^b,c^ Results are presented only for interactions or predictors with raw *p*-values < 0.05; after the false discovery rate adjustment, the interaction/predictor *p*-values were considered to be significant at <0.032 for total protein concentration (none are significant), at <0.046 for casein-whey ratio (none are significant), and at <0.05 for casein and whey protein concentrations (none are significant, data not shown).

**Table A3:** Associations between calculated daily intakes of casein and whey protein and infant characteristics.

Predictor (CDI ^d^, g)	5 Months		9 Months		12 Months			*p*-Value	
	Intercept (SE)	Slope (SE)	Intercept (SE)	Slope (SE)	Intercept (SE)	Slope (SE)	Predictor	Infant Age (months)	Interaction
	***n =* 17**		***n =* 7**		***n =* 8**		***n =* 18**		
**Infant Fat Mass with Ultrasound 2 Skinfolds (kg)**									
Whey protein	1.31 (0.30) ^a^	0.148 (0.062)	1.98 (0.23)	0.148 (0.062)	2.03 (0.24)	0.148 (0.062)	0.024 ^b^	<0.001	0.56 ^c^
**Infant fat mass with ultrasound 2 skinfolds (%)**									
Whey protein1	20.60 (2.65)	1.330 (0.554)	22.90 (2.12)	1.330 (0.554)	21.60 (2.03)	1.330 (0.554)	0.033	0.62	0.53
	***n =* 17**		***n =* 7**		***n =* 7**		***n =* 18**		
**Infant Head Circumference (cm)**									
Casein	43.10 (0.45)	−0.243 (0.194)	44.70 (0.55)	1.330 (0.680)	46.80 (0.46)	−0.334 (0.490)	0.23	<0.001	0.046
**Infant Body Mass Index (kg/m^2^)**									
Casein	16.70 (0.65)	0.676 (0.358)	18.30 (0.98)	−0.788 (1.430)	18.80 (0.74)	−2.10 (1.040)	0.33	0.59	0.047
**Infant Fat-Free Mass Index with Bioelectrical Impedance Spectroscopy (kg/m^2^)**									
Whey protein	14.10 (0.61)	−0.319 (0.121)	14.70 (0.48)	−0.319 (0.121)	14.30 (0.50)	−0.319 (0.121)	0.004	0.075	0.20
**Infant Fat Mass Index with Ultrasound 2 Skinfolds (kg/m^2^)**									
Casein	3.99 (0.42)	0.517 (0.248)	4.55 (0.36)	0.517 (0.248)	4.15 (0.35)	0.517 (0.248)	0.048	0.32	0.77
Whey protein	3.22 (0.65)	0.336 (0.135)	3.88 (0.51)	0.336 (0.135)	3.42 (0.52)	0.336 (0.135)	0.016	0.20	0.73
	***n =* 16**		***n =* 7**		***n =* 8**		***n =* 18**		
**Infant Fat-Free Mass with Ultrasound 4 Skinfolds (kg)**									
Casein	6.08 (0.26)	−0.456 (0.144)	6.77 (0.21)	−0.456 (0.144)	7.68 (0.21)	−0.456 (0.144)	**0.003**	<0.001	0.79
**Infant Fat Mass with Ultrasound 4 Skinfolds (kg)**									
Casein	1.38 (0.18)	0.390 (0.100)	2.14 (0.15)	0.390 (0.100)	1.99 (0.14)	0.390 (0.100)	**<0.001**	<0.001	0.28
**Infant Fat Mass with Ultrasound 4 Skinfolds (%)**									
Casein	20.30 (1.62)	4.220 (0.932)	24.40 (1.31)	4.220 (0.932)	20.60 (1.26)	4.220 (0.932)	**<0.001**	0.002	0.31
Whey protein	21.30 (2.65)	1.190 (0.565)	23.60 (2.18)	1.190 (0.565)	20.10 (2.05)	1.190 (0.565)	0.038	0.16	0.62
	***n =* 16**		***n =* 7**		***n =* 7**		***n =* 18**		
**Infant Fat-Free Mass Index with Ultrasound 4 Skinfolds (kg/m^2^)**									
Casein	13.70 (0.43)	−0.484 (0.235)	13.20 (0.61)	−0.119 (0.901)	14.90 (0.47)	−2.28 (0.662)	0.001	0.021	0.022
**Infant Fat Mass Index with Ultrasound 4 Skinfolds (kg/m^2^)**									
Casein	3.57 (0.42)	0.789 (0.240)	4.35 (0.34)	0.789 (0.240)	3.55 (0.34)	0.789 (0.240)	**0.001**	0.013	0.55
Whey protein	3.39 (0.67)	0.309 (0.143)	3.94 (0.55)	0.309 (0.143)	3.07 (0.55)	0.309 (0.143)	0.034	0.12	0.74

^a^ Parameter estimate ± SE; effects of predictors taken from linear mixed effects models that accounted for month after birth and an interaction between month after birth and predictor with a random effect for participant; if the interaction is not significant parameter estimates are taken from a model with no interaction. ^b,c^ Results are presented only for interactions or predictors with raw *p*-values < 0.05; after the false discovery rate adjustment interaction/predictor *p*-values were considered to be significant at <0.022 for calculated daily intakes (CDI) of casein (indicated by the bold text), at <0.004 for CDI of whey protein (none are significant) and at <0.05 for CDI of total protein (none are significant, data not shown). ^d^ CDI were measured between 2 and 5 months and within 2 weeks of 9 and 12 months.

**Table A4:** Associations between feeding frequency and human milk proteins.

Predictor (Concentration of Proteins or Breastfeeding Parameter)	2 Months		5 Months			9 Months		12 Months			*p*-Value	
	Intercept (SE)	Slope (SE)	Intercept (SE)	Slope (SE)	Intercept (SE)	Slope (SE)	Intercept (SE)	Slope (SE)		Predictor	Infant Age (Months)	Interaction
	***n =* 11**		***n =* 19**			***n =* 17**		***n =* 11**		***n =* 19**		
**Infant Feeding Frequency (Self-Reported as Hours between Feeds) ^e^**												
Total protein (g/L)	3.97 (0.94) ^a^	−0.158 (0.078)	4.56 (0.95)	−0.158 (0.078)		5.29 (0.87)	−0.158 (0.078)	7.16 (0.94)	−0.158 (0.078)	0.039 ^b^	<0.001	0.30 ^c^
	***n/a*^d^**		***n =* 17**			***n =* 8**		***n =* 8**		***n =* 18**		
**CDI of Total Protein (g)**												
Feeding frequency (24-h MP) ^d^	n/a ^d^	n/a ^d^	2.15 (2.68)	0.317 (0.004)		0.87 (1.92)	0.317 (0.004)	1.34 (1.69)	0.317 (0.004)	**0.004**	0.41	0.22
Feeding frequency (SR) ^e^	n/a	n/a	11.10 (0.97)	0.19 (0.002)		7.93 (1.17)	0.19 (0.002)	9.15 (1.51)	0.19 (0.002)	**0.003**	<0.001	0.90
**CDI of Casein (g) ^d^**												
Feeding frequency (24-h MP)	n/a	n/a	0.01 (0.62)	0.074 (0.017)		−0.34 (0.44)	0.074 (0.017)	−0.18 (0.02)	0.074 (0.017)	**0.024**	0.36	0.54
Feeding frequency (SR)	n/a	n/a	1.81 (0.22)	0.054 (0.014)		1.19 (0.29)	0.054 (0.014)	1.54 (0.40)	0.054 (0.014)	**0.012**	0.005	0.57
**CDI of Whey Protein (g) ^d^**												
Feeding frequency (24-h MP)	n/a	n/a	1.39 (1.10)	0.131 (0.003)		0.95 (0.78)	0.131 (0.003)	1.30 (0.68)	0.131 (0.003)	**0.003**	0.36	0.084
Feeding frequency (SR)	n/a	n/a	5.16 (0.39)	0.095 (0.017)		3.88 (0.52)	0.095 (0.017)	4.50 (0.71)	0.095 (0.017)	**0.011**	<0.001	0.25

^a^ Parameter estimate ± SE; effects of predictors taken from linear mixed effects models that accounted for month after birth and an interaction between month after birth and predictor with a random effect for participant; if the interaction is not significant parameter estimates are taken from a model with no interaction. ^b,c^ Results are presented only for interactions or predictors with raw *p*-values < 0.05; after the false discovery rate adjustment interaction/predictor *p*-values were considered to be significant at <0.039 for self-reported feeding frequency and concentrations of the proteins, at <0.05 for 24-h milk production (MP) feeding frequency and concentrations of the proteins (none are significant, data not shown), at <0.05 for calculated daily intakes (CDI) of total protein, casein and whey protein and both FFQ (indicated by the bold text). ^d^ CDI, 24-h milk intake and 24-h milk production (24-h MP) feeding frequency were measured between 2 and 5 months (presented at 5 months here) and within 2 weeks of 9 and 12 months; n/a–not applicable. ^e^ Feeding frequency was self-reported (SR) by mothers at the time of the visit as an average time between meals (e.g., each 2 h).

**Table A5:** Associations between calculated daily intakes of human milk total protein at given time points and changes in infant body composition between the time points.

Changes in Infant Characteristics between Time Points	Months after Birth					
	5 and 2 Months	9 and 2 Months	12 and 2 Months	9 and 5 Months	12 and 5 Months	12 and 9 Months
**CDI of Total Protein (g) between 2 and 5 Months (*n* = 17) ^e^**						
ΔHead circumference (cm)	−0.14 (0.14) ^a^	0.03 (0.17)	0.14 (0.16)	−0.04 (0.05)	0.03 (0.03)	**0.09 (0.04)**
	0.35 ^b, c^	0.88	0.43	0.44	0.33	**0.046**
ΔLength (cm)	−0.08 (0.17)	**0.40 (0.15)**	**0.57 (0.17)**	−0.01 (0.11)	0.15 (0.13)	0.21 (0.12)
	0.62	**0.025**	**0.011**	0.91	0.29	0.12
ΔWeight (kg)	0.09 (0.05)	**0.18 (0.07)**	0.17 (0.09)	0.03 (0.03)	0.03 (0.04)	0.01 (0.01)
	0.11	**0.030**	0.096	0.36	0.42	0.50
ΔBMI ^d^ (kg/m^2^)	**0.29 (0.09)**	0.13 (0.16)	0.06 (0.21)	0.03 (0.08)	−0.05 (0.10)	−0.09 (0.05)
	**0.013**	0.42	0.79	0.67	0.62	0.13
ΔFat-free mass US 4SF ^d^ (kg)	0.04 (0.07)	**0.19 (0.07)**	0.18 (0.12)	0.004 (0.03)	0.03 (0.04)	0.03 (0.02)
	0.55	**0.037**	0.19	0.89	0.46	0.13
ΔFat-free mass BIS ^d^ (kg)	0.05 (0.03)	0.13 (0.09)	**0.18 (0.07)**	0.02 (0.03)	0.08 (0.04)	**0.08 (0.03)**
	0.13	0.15	**0.041**	0.60	0.065	**0.041**
ΔFat-free mass index US 4SF (kg/m^2^)	**0.27 (0.09)**	0.18 (0.14)	0.39 (0.22)	0.01 (0.05)	−0.02 (0.07)	−0.02 (0.06)
	**0.023**	0.25	0.12	0.86	0.81	0.71
ΔFat-free mass index BIS (kg/m^2^)	**0.14 (0.06)**	0.11 (0.15)	0.11 (0.15)	0.04 (0.06)	0.04 (0.07)	0.03 (0.06)
	**0.042**	0.46	0.49	0.51	0.59	0.59
ΔFat mass US 2SF (kg/m^2^)	**0.13 (0.06)**	0.07 (0.05)	0.12 (0.08)	0.03 (0.03)	0.004 (0.03)	−0.02 (0.01)
	**0.049**	0.22	0.16	0.43	0.91	0.25
ΔFat mass index US 2SF (kg/m^2^)	0.26 (0.13)	0.07 (0.12)	0.09 (0.19)	0.04 (0.08)	−0.03 (0.08)	**−0.07 (0.03)**
	0.081	0.58	0.64	0.58	0.70	**0.029**
ΔFat mass index BIS (kg/m^2^)	**0.17 (0.07)**	0.04 (0.15)	−0.03 (0.14)	−0.003 (0.07)	−0.13 (0.08)	−0.14 (0.07)
	**0.044**	0.82	0.84	0.97	0.12	0.075
**CDI of Total Protein (g) at 9 Months (*n* = 8) ^e^**						
ΔHead circumference (cm)	n/a ^f^	−0.54 (0.22)	0.04 (0.33)	0.13 (0.09)	−0.17 (0.10)	**−0.30 (0.09)**
		0.14	0.91	0.21	0.14	**0.021**
ΔFat-free mass index BIS (kg/m^2^)	n/a	**−0.36 (0.02)**	0.07 (0.43)	−0.20 (0.12)	−0.12 (0.16)	0.08 (0.20)
		**0.004**	0.89	0.14	0.47	0.70
**CDI of Total Protein (g) at 12 Months (*n* = 8) ^e^**						
ΔBMI ^d^ (kg/m^2^)	n/a ^f^	n/a ^f^	0.08 (0.32)	n/a ^f^	−0.24 (0.25)	**−0.30 (0.09)**
			0.84		0.40	**0.021**
ΔFat-free mass index US 2SF (kg/m^2^)	n/a	n/a	−0.32 (0.30)	n/a	**−0.48 (0.18)**	−0.20 (0.12)
			0.39		**0.045**	0.17
ΔFat-free mass index US 4SF (kg/m^2^)	n/a	n/a	−0.30 (0.38)	n/a	**−0.50 (0.09)**	−0.21 (0.17)
			0.51		**0.006**	0.26
ΔFat mass US 2SF (kg)	n/a	n/a	**0.21 (0.05)**	n/a	**0.14 (0.06)**	−0.001 (0.04)
			**0.024**		**0.043**	0.99
ΔFat mass US 2SF (%)	n/a	n/a	2.66 (0.93)	n/a	**1.60 (0.59)**	−0.17 (0.55)
			0.064		**0.036**	0.77
ΔFat mass index US 2SF (kg/m^2^)	n/a	n/a	**0.38 (0.08)**	n/a	0.25 (0.14)	−0.11 (0.10)
			**0.045**		0.13	0.33

^a^ Parameter estimates ± SE and ^b^ *p*-values for associations between calculated daily intakes (CDI) of casein at given time points and the changes (Δ) in measured variables between different months after birth. ^c^ Results are presented only for variables with at least one significant raw *p*-value (*p* < 0.05, indicated by the bold text); after the false discovery rate adjustment, the predictor *p*-values were considered to be significant at <0.011 for CDI of total protein between 2 and 5 months, at <0.004 for CDI at 9 months and at <0.006 for CDI at 12 months (none are significant). ^d^ BIS—bioelectrical impedance spectroscopy; BMI—body mass index; US 2SF—ultrasound 2-skinfolds; US 4SF—ultrasound 4-skinfolds. ^e^ CDI were measured between 2 and 5 months and within 2 weeks of 9 and 12 months. ^f^ Results are not presented for impractical time combinations, n/a–not applicable.

**Table A6:** Associations between calculated daily intakes of human milk casein at given time points and changes in infant body composition between the time points.

Changes in Infant Characteristic	Months after Birth					
	5 and 2	9 and 2	12 and 2	9 and 5	12 and 5	12 and 9
**CDI of Casein (g) between 2 and 5 Months (*n* = 17) ^e^**						
ΔHead circumference (cm)	**−1.17 (0.39) ^a^ 0.016 ^b,c^**	−0.79 (0.60)	−0.68 (0.58)	0.01 (0.22)	0.17 (0.15)	0.23 (0.22)
		0.23	0.28	0.97	0.29	0.31
ΔFat mass US 4SF (kg)	0.02 (0.10)	**−0.47 (0.17)**	**−0.49 (0.20)**	−0.07 (0.15)	−0.17 (0.13)	−0.06 (0.07)
	0.83	**0.031**	**0.040**	0.67	0.21	0.40
ΔFat mass US 2SF (%)	2.94 (3.17)	**−3.62 (1.50)**	−2.02 (3.41)	−0.05 (1.76)	−1.37 (1.66)	−1.01 (0.68)
	0.38	**0.042**	0.57	0.98	0.42	0.16
ΔFat mass US 4SF (%)	0.35 (1.53)	**−6.67 (1.36)**	**−6.27 (2.42)**	−1.18 (1.60)	−2.51 (1.31)	−0.92 (0.79)
	0.83	**0.002**	**0.036**	0.47	0.078	0.27
ΔFat mass BIS (%)	0.94 (1.36)	−4.12 (2.78)	**−4.48 (1.61)**	0.24 (1.51)	−0.77 (1.63)	−1.48 (1.73)
	0.51	0.18	**0.027**	0.88	0.64	0.41
ΔFat mass index US 4SF (kg/m^2^)	0.001 (0.27)	**−1.29 (0.32)**	**−1.44 (0.50)**	−0.23 (0.36)	−0.48 (0.33)	−0.20 (0.15)
	1.00	**0.005**	**0.028**	0.53	0.18	0.19
**CDI of Casein (g) at 9 Months (*n* = 8) ^e^**						
ΔHead circumference (cm)	n/a ^f^	3.33 (3.00)	−1.04 (2.72)	0.62 (0.79)	**−1.78 (0.62)**	**−2.40 (0.74)**
		0.38	0.74	0.47	**0.036**	**0.023**
**CDI of Casein (g) at 12 Months (*n* = 8) ^e^**						
ΔHead circumference (cm)	n/a ^f^	n/a ^f^	**4.01 (0.60)**	n/a ^f^	−0.97 (0.55)	−0.66 (1.11)
			**0.022**		0.14	0.58
ΔBMI ^d^ (kg/m^2^)	n/a	n/a	−1.36 (3.92)	n/a	−2.33 (1.57)	**−2.09 (0.61)**
			0.76		0.20	**0.019**
ΔFat−free mass US 4SF ^d^ (kg)	n/a	n/a	**−3.77 (0.73)**	n/a	−1.05 (0.83)	−0.51 (0.53)
			**0.014**		0.26	0.38
ΔFat−free mass index US 2SF (kg/m^2^)	n/a	n/a	−5.39 (2.60)	n/a	−2.99 (1.40)	**−2.13 (0.45)**
			0.17		0.086	**0.005**
ΔFat−free mass index US 4SF (kg/m^2^)	n/a	n/a	−7.12 (1.69)	n/a	**−2.84 (0.87)**	**−2.40 (0.78)**
			0.052		**0.031**	**0.028**
ΔFat mass US 4SF (%)	n/a	n/a	**33.56 (8.59)**	n/a	6.08 (7.30)	4.85 (4.71)
			**0.030**		0.44	0.35

^a^ Parameter estimates ± SE and ^b^ *p*-values for associations between calculated daily intakes (CDI) of whey protein at given time points and the changes (Δ) in measured variables between different months after birth. ^c^ Results are presented only for variables with at least one significant raw *p*-value (*p* < 0.05, indicated by the bold text); after the false discovery rate adjustment, the predictor *p*-values were considered to be significant at <0.002 for CDI of casein between 2 and 5 months, at <0.023 for CDI at 9 months and at <0.005 for CDI at 12 months (none are significant). ^d^ BIS—bioielctrical impedance spectroscopy; BMI—body mass index; US 2SF—ultrasound 2-skinfolds; US 4SF—ultrasound 4-skinfolds. ^e^ CDI were measured between 2 and 5 months and within 2 weeks of 9 and 12 months. ^f^ Results are not presented for impractical time combinations, n/a, not applicable.

**Table A7:** Associations between calculated daily intakes of total human milk whey protein at given time points and changes in infant body composition between the time points.

Changes in Infant Characteristic	Months after Birth					
	5 and 2	9 and 2	12 and 2	9 and 5	12 and 5	12 and 9
**CDI of Whey Protein (g) between 2 and 5 Months (*n* = 17) ^e^**						
ΔLength (cm)	−0.24 (0.33) ^a^ 0.49 ^b,c^	0.44 (0.39)	**1.01 (0.35)**	−0.06 (0.31)	0.17 (0.40)	0.37 (0.38)
		0.29	**0.024**	0.85	0.67	0.35
ΔBMI ^d^ (kg/m^2^)	**0.58 (0.19)**	0.31 (0.31)	−0.02 (0.40)	−0.02 (0.22)	−0.23 (0.28)	−0.21 (0.16)
	**0.017**	0.35	0.97	0.94	0.42	0.20
ΔFat-free mass index US 4SF (kg/m^2^)	**0.65 (0.21)**	0.20 (0.35)	0.12 (0.55)	0.003 (0.16)	−0.15 (0.21)	−0.05 (0.17)
	**0.016**	0.58	0.83	0.99	0.50	0.77
ΔFat mass US 2SF (kg)	**0.29 (0.12)**	0.15 (0.10)	0.26 (0.15)	0.01 (0.10)	−0.01 (0.10)	0.01 (0.04)
	**0.032**	0.18	0.12	0.96	0.89	0.89
ΔFat mass index US 2SF (kg/m^2^)	**0.61 (0.25)**	0.21 (0.23)	0.25 (0.36)	−0.03 (0.22)	−0.15 (0.24)	−0.09 (0.10)
	**0.042**	0.38	0.51	0.88	0.54	0.38
**CDI of Whey Protein (g) at 12 Months (*n* = 8) ^e^**						
ΔFat-free mass index US 4SF (kg/m^2^)	n/a ^f^	n/a ^f^	−0.18 (0.57)	n/a ^f^	**−0.62 (0.22)**	−0.10 (0.31)
			0.78		**0.049**	0.76
ΔFat mass US 2SF (kg)	n/a	n/a	**0.26 (0.06)**	n/a	0.13 (0.11)	−0.01 (0.06)
			**0.025**		0.27	0.92

^a^ Parameter estimates ± SE and ^b^ *p*-values for associations between calculated daily intakes (CDI) of total protein at given time points and the changes (Δ) in measured variables between different months after birth. ^c^ Results are presented only for variables with at least one significant raw *p*-value (*p* < 0.05, indicated by the bold text); after the false discovery rate adjustment, the predictor *p*-values were considered to be significant at < 0.016 for CDI of casein between 2 and 5 months, at < 0.05 for CDI at 9 months and at <0.025 for CDI at 12 months (none are significant). ^d^ BIS—bioielectrical impedance spectroscopy; BMI—body mass index; US 2SF—ultrasound 2-skinfolds; US 4SF—ultrasound 4-skinfolds. ^e^ CDI were measured between 2 and 5 and within 2 weeks of 9 (none are significant, data not shown) and 12 months. ^f^ Results are not presented for impractical time combinations, n/a–not applicable.
